# Lymphocyte morphology in a patient receiving CD19 chimeric antigen receptor T‐cell therapy for mantle cell lymphoma

**DOI:** 10.1002/jha2.334

**Published:** 2021-11-18

**Authors:** Maria A. V. Marzolini, Lorna Neill, Maeve O'Reilly, Karl S. Peggs, Claire Roddie

**Affiliations:** ^1^ Department of Haematology University College London Hospitals NHS Foundation Trust London UK

A 56‐year‐old male with a diagnosis of relapsed mantle cell lymphoma was recruited to a clinical trial to receive CD19 chimeric antigen receptor (CAR) T‐cell therapy (ALLCAR19 trial, NCT02935257). He had previously been treated with multiple lines of therapy including both an autologous and allogeneic haematopoietic stem cell transplant, Ibrutinib and radiotherapy. Prior to commencing lymphodepletion, his bone marrow biopsy showed 25% infiltration with residual lymphoma. He received a dose of 200 × 10^6^ CAR T cells and tolerated the infusion well. On Day +1, he was pyrexial and was treated for neutropenic fevers with intravenous antibiotics. He was also diagnosed with Grade 1 cytokine release syndrome. He received tocilizumab on Day +6 and subsequently became apyrexial.

On Day +9, he developed a lymphocytosis of 3.74 × 10^9^/L (Normal range 1.2–3.65 × 10^9^/L) and remained neutropenic (0.02 × 10^9^/L) with a total white cell count of 4.15 × 10^9^/L. He was thrombocytopenic with a platelet count of 26 × 10^9^/L and his haemoglobin was 103 g/L. The differential diagnoses for his lymphocytosis included disease relapse, infection or peripheral blood CAR T‐cell expansion.

**FIGURE 1:**
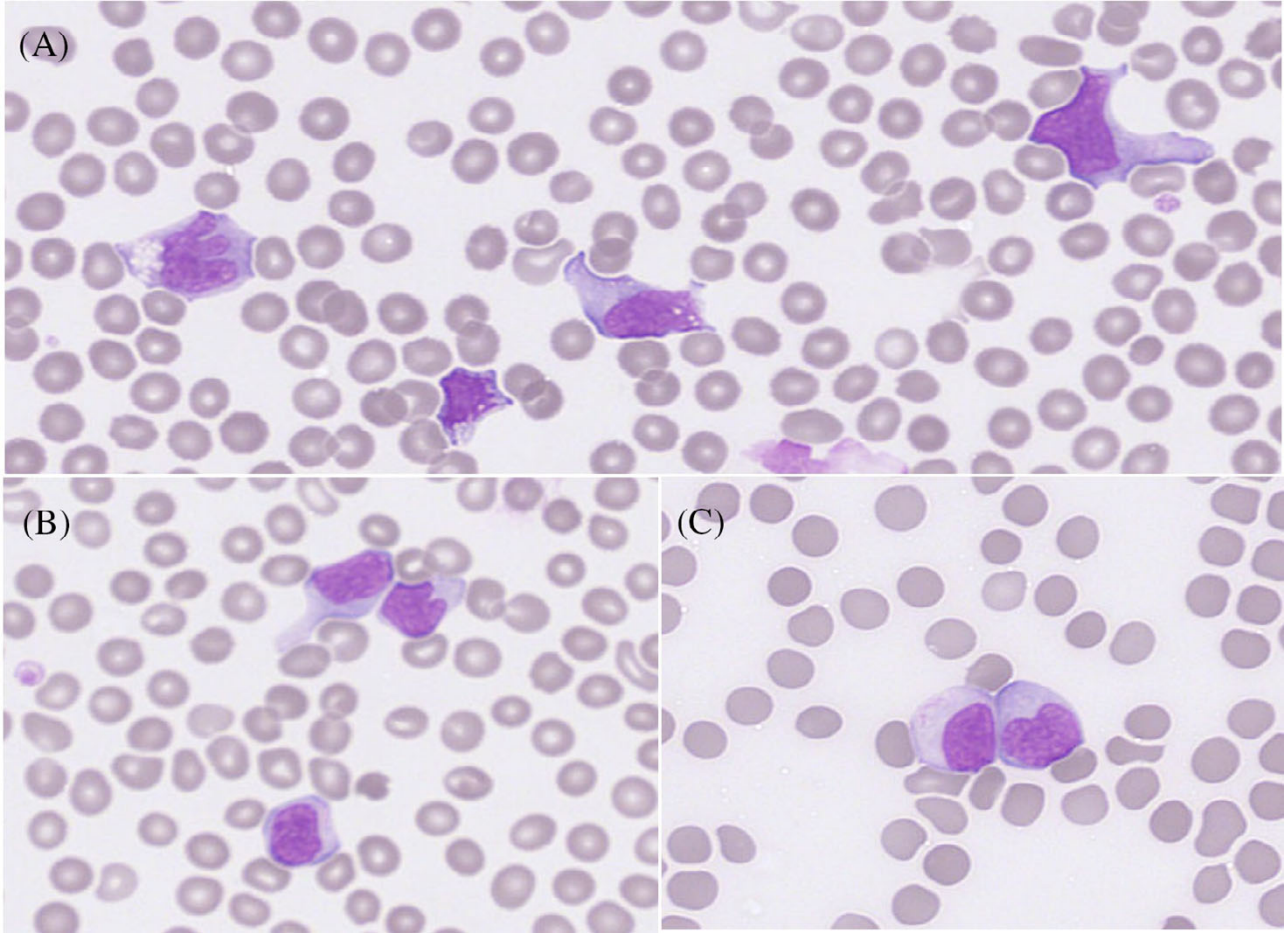
The peripheral blood film showing numerous atypical lymphocytes with abundant cytoplasm, vacuolation (A) and clefted nuclei (B and C). Images taken at 40× magnification

His peripheral blood film demonstrated frequent atypical lymphocytes with clefted nuclei, abundant cytoplasm and occasional vacuolation (Figure 1). Lymphocyte subsets demonstrated 97.3% were CD3+ positive with a low CD4:CD8 ratio of 0.17. Further flow cytometry studies confirmed that 88% of the peripheral blood lymphocytes were CAR‐T cells with an absolute number of 4954 CAR‐T cells/μl.

This case demonstrates the morphology of peripheral blood CAR T‐cells, which appear as atypical lymphocytes. Patients receiving CAR‐T cell therapy may develop a temporary lymphocytosis as the CAR‐T cells are proliferating. It is important that this is differentiated from other causes of a lymphocytosis such as infection or disease relapse and haematologists should be aware of the morphological features of these cells.

